# LncRNA MEG3 rs3087918 was associated with a decreased breast cancer risk in a Chinese population: a case-control study

**DOI:** 10.1186/s12885-020-07145-0

**Published:** 2020-07-15

**Authors:** Yi Zheng, Meng Wang, Shuqian Wang, Peng Xu, Yujiao Deng, Shuai Lin, Na Li, Kang Liu, Yuyao Zhu, Zhen Zhai, Ying Wu, Zhijun Dai, Gaixia Zhu

**Affiliations:** 1 Department of Obstetrics and Gynecology, The Second Affiliated Hospital of Xi’an Jiaotong University, Xi’an, 710004 China; 2 Department of Breast Surgery, The First Affiliated Hospital, College of Medicine, Zhejiang University, Hangzhou, 310003 China; 3 Department of Oncology, The Second Affiliated Hospital of Xi’an Jiaotong University, Xi’an, 710004 China; 4 Department of Hepatobiliary Surgery, The First Affiliated Hospital of Xi’an Jiaotong University, Xi’an, 710061 China

**Keywords:** MEG3, SNP, Breast cancer, Case-control study, miRNA

## Abstract

**Background:**

LncRNA MEG3 expressed abnormally in various cancers including breast cancer, but no studies reported the correlation between MEG3 SNPs and breast cancer susceptibility among Chinese women.

**Methods:**

This study is aimed to explore the association between three SNPs of MEG3 (rs3087918, rs7158663, rs11160608) and breast cancer. The study is a population-based case-control study including 434 breast cancer patients and 700 healthy controls. Genotyping was performed using Sequenom MassArray technique. Function prediction of rs3087918 were based on RNAfold and lncRNASNP2 databases.

**Results:**

Pooled analysis indicated that rs3087918 was related to a decreased risk of breast cancer [GG vs. TT: OR (95%) = 0.67(0.45–0.99), *P* = 0.042; GG vs. TT + TG: OR (95%) = 0.69(0.48–0.99), *P* = 0.046], especially for women aged <=49 [GG vs. TT: OR (95%) = 0.40(0.22–0.73), *P* = 0.02]. Comparison between case groups showed genotype GG and TG/GG of rs3087918 were associated with her-2 receptor expression [GG vs. TT: OR (95%) = 2.37(1.24–4.63), *P* = 0.010; TG + GG vs. TT: OR (95%) = 1.50(1.01–2.24), *P* = 0.045]. We didn’t find statistical significance for rs11160608, rs7158663 and breast cancer. Structure prediction based on RNAfold found rs3087918 may influence the secondary structure of MEG3. The results based on lncRNASNP2 indicated that rs3087918 may gain the targets of hsa-miR-1203 to MEG3, while loss the target of hsa-miR-139-3p and hsa-miR-5091 to MEG3.

**Conclusions:**

MEG3 rs3087918 was associated with a decreased risk of breast cancer. MEG3 haplotype TCG may increase the risk of breast cancer.

## Background

Breast cancer (BC) is a serious threat to women’s health. According to American *cancer statistics 2020* [1], there will be an estimated 276,480 new BC cases and 42,170 BC related death in 2020. For females, BC is the most common diagnosed cancer (24.2% of the total cases) and the leading cause of cancer death (15.0% of the total cancer death). Although epidemiological studies have identified several risk factors involved in BC, such as age, hormonal state, and family history [2], the pathogenesis of BC is still unclear. BC is a complex and genetically heterogeneous disease in which genetic changes such as abnormal amplification of oncogenes, or deletion/mutation of tumor suppressor genes, play a substantial role [3–5].

Maternally expressed gene 3 (MEG3) is an imprinted gene located at chromosome 14q32.3 in humans, encoding a long non-coding RNA (lncRNA) belonging to the imprinted DLK1-MEG3 regions [6]. This region contains at least three paternally expressed protein coding genes and numerous maternally expressed noncoding RNAs [7]. The imprinted expression of these genes was related to cell development and growth [8], and experiments in vitro indicated MEG3 can suppress the proliferation of human cancer cells lines [9]. Researchers found loss of MEG3 related to a variety of human cancers, such as gastric [10], cervical [11], and breast [12] cancer. MEG3 can inhibit the occurrence of tumor through various aspects. Firstly, MEG3 can inhibit the proliferation of tumor cells and consequently induce apoptosis, which has been confirmed by in vitro experiments and animal models [13]. Secondly, MEG3 plays a role in epigenetic regulation and can alter the function of cancer cells by affecting DNA methylation and regulating the functions of snoRNA and miRNA [14, 15]. Moreover, MEG3 is involved in the regulation of many tumor-related signaling pathways, including p53, MDM2, and pRb pathway [16].

Single-nucleotide polymorphism (SNP) mainly refers to the DNA sequence polymorphism caused by the variation of a single nucleotide at the genome level. It is the most common genetic variant in the human genome, accounting for 90% of all known polymorphisms [17]. To date, Genome Wide Association Study (GWAS) and multiple large-scale sequencing have identified many SNPs in more than 70 genes associated with breast cancer [18, 19]. SNP has been considered a potential biomarker of genetic background to predict risk, progression, and treatment response to various diseases. Previous investigation indicated that several SNPs in MEG3 genes are associated with breast cancer susceptibility [20]. However, there are no investigation to explore the relationship between MEG3 polymorphisms and breast cancer among Chinese women. In this study, we genotyped three polymorphisms (rs3087918, rs11160608 rs7158663) in MEG3 gene based on 434 BC patients and 700 healthy controls, to explore their relationship with breast cancer.

## Methods

### Study subjects

In total, 1134 females were recruited for this population-based case-control study. Among these, 434 breast cancers were enrolled in the Department of Oncology, the Second Affiliated Hospital, Xi’an Jiaotong University, from 2013 to 2015. Seven hundred healthy females were randomly recruited from medical center of the same hospital during the same period. All BC patients were diagnosed by pathology and detailed immunohistochemical analysis. BC patients who had a history of other malignant diseases or receiving chemotherapy or radiotherapy were excluded. The controls were matched to cases by age (±2 years) and had no history of malignant tumors, no history of chemoradiotherapy, no obvious abnormality in blood routine examination. The protocol of this study was approved by the Ethics Committee of the Second Affiliated Hospital of Xi’an Jiaotong University Shaanxi Province (Xi’an, China). All patients gave written informed consent prior to participation in the study.

### SNP selection and genotyping

SNPs were selected from NCBI dbSNP database (https://www.ncbi.nlm.nih.gov/projects/SNP) and relevant literature [20–22] according to the following criteria. First, the minor allele frequency (MAF) was no less than 0.05 among Chinese population. Secondly, the SNPs located in the 5′- flanking region, 5′ untranslated region, 3′ untranslated region, and exon of MEG3 gene. We finally chose three MEG3 SNPs rs3087918, rs11160608 rs7158663 to study. Peripheral blood samples were collected in EDTA-coated tubes and conserved at − 80 °C. Genome DNA were extracted from whole blood samples using ComWin BloodGen Mini Kit (QIAGEN, China, Beijing). Ultraviolet spectrophotometer (Nanodrop, Thermo Scientific, Waltham, MA) was utilized to measure the purity and concentration of extracted DNA. We designed multiplexed SNP MassEXTEND assay using Sequenom MassARRAY Assay Design 3.0 software. DNA samples were genotyped by Sequenom MassARRAY RS1000 according to the standard protocol. The primers applied for the three SNPs were shown in Supplemental Table S1.

### Statistical analysis

The HWE of the three SNPs were calculated using Fisher’s exact test in controls group. Student’s t test was adopted to evaluated the difference of age distribution and body mass index (BMI) between BC patients and healthy controls. Two-sided Pearson’s chi-square tests were applied to access the differences in the categorical variables between cases and controls, such as age (<=49 and > 49), BMI, menstrual-status, and allelic frequencies. *P* < 0.05 was considered statistically significant. We also calculated odds ratios (ORs) and 95% confidence intervals (CIs) using logistic regression analysis. Haplotype analysis were conducted by Haploview 4.2. Other statistical analyses were performed using the version R 3.5.2 software.

### Function prediction based on databases

We used RNAfold (http://rna.tbi.univie.ac.at//cgi-bin/RNAWebSuite/RNAfold.cgi) and LncRNASNP2 (http://bioinfo.life.hust.edu.cn/lncRNASNP/) database to predict the effect of SNP on MEG3. RNAfold is a classic database to predict RNAs structure. Free energy represents the amount of energy that needs to be injected to change the structure. The smaller the corresponding value is, the more stable the structure will be. LncRNASNP2 is a novel database containing 7,260,238 SNPs on 141,353 human lncRNA transcripts and 3,921,448 SNPs on 117,405 mouse lncRNA transcripts [23]. We used this database to predict the potential function of the MEG3 polymorphisms.

## Results

### Demographical and clinical information of study population

This study contained 434 BC cases and 700 healthy control. All the subjects were Han Chinses women from northwest China. There were no statistically significant differences in age distribution, BMI and menopausal status between the patients and the control group. The detail demographical and clinical information was display in Table 1. BMI was a statistical index to estimate the body fat in people of any age. In this study, BMI was divided into four levels (underweight, normal weight, overweight, and obese) based on Chinese reference standard.

**Table 1:** Demographic information

Characteristics		Cases (%)	Controls (%)	*P* value
Number		434	700	
Age (mean ± SD)		51.95 ± 10.35	51.83 ± 17.28	0.879^a^
≦49		180 (41.5)	298 (42.6)	
>49		254 (58.5)	402 (57.4)	0.716
BMI, kg/m2 (mean ± SD)		22.38 ± 2.61	22.71 ± 4.00	0.084^a^
Menopausal status				
Premenopausal		157 (36.2)	188 (41.8)	
Postmenopausal		277 (63.8)	262 (58.2)	0.506
TNM Stage				
I		114 (26.3)	–	–
II		192 (44.2)		–
III		89 (20.5)	–	–
IV		39 (9)	–	–
Immunohistochemistry results				
ER	–	142 (32.7)	–	–
	+	292 (67.3)	–	–
PR	–	189 (43.5)	–	–
	+	245 (56.5)	–	–
Her-2	–	250 (57.6)	–	–
	+	184 (42.4)	–	–

*BMI:* body mass index, *ER:* estrogen receptor, *PR:* progesterone receptor, *Her-2:* human epidermal growth factor receptor-2

^a^ Student’s t-test

### The associations between *MEG3* SNPs and BC risk

Three SNP in MEG3 gene (rs3087918, rs11160608 rs7158663) were genotyped in all recruited subjects, and their detected rate were 99.1, 99.2 and 99.4%, respectively. The genotype distribution of the three polymorphisms in control groups accorded with HWE (rs11160608: *P*_*HWE*_ = 0.844; rs3087918: *P*_*HWE*_ = 0.968; rs7158663: *P*_*HWE*_ = 0.334). We didn’t find statistical significance for rs11160608, rs7158663 and breast cancer (*P* > 0.05 in all genetic models). Pooled analysis indicated that rs3087918 was related to a decreased risk of breast cancer [GG vs. TT: OR (95%CI) = 0.67(0.45–0.99), *P* = 0.042; GG vs. TT + TG: OR (95% CI) = 0.69(0.48–0.99), *P* = 0.046]. The detail results were showed in Table 2.

**Table 2:** Association between MEG3 gene polymorphisms and risk of breast cancer (rs11160608, rs3087918, rs7158663)

SNPs genetic model	Genotype	Cases (%) *N* = 434	Controls (%) *N* = 700	OR (95%CI)	*P* value
rs11160608					
Co-dominant	AA	126 (29.7)	227 (32.4)	reference	
	AC	218 (51.4)	341 (48.7)	1.15 (0.87–1.52)	0.316
	CC	80 (18.9)	132 (18.9)	1.09 (0.77–1.55)	0.625
Dominant	AA	126 (29.7)	227 (32.4)	reference	
	AC + CC	298 (70.3)	473 (67.6)	1.14 (0.87–1.48)	0.342
Recessive	AA+AC	344 (81.1)	568 (81.1)	reference	
	CC	80 (18.9)	132 (18.9)	1.00 (0.74–1.36)	0.996
Allele	A	470 (55.4)	795 (56.8)	reference	
	C	378 (44.6)	605 (43.2)	1.06 (0.89–1.26)	0.528
rs3087918					
Co-dominant	TT	171 (40.2)	259 (37.0)	reference	
	TG	207 (48.7)	334 (47.7)	0.94 (0.72–1.22)	0.633
	GG	47 (11.1)	107 (15.3)	0.67 (0.45–0.99)	0.042*
Dominate	TT	171 (40.2)	259 (37.0)	reference	
	TG + GG	254 (59.8)	441 (63.0)	0.87 (0.68–1.12)	0.279
Recessive	TT + TG	378 (88.9)	593 (84.7)	reference	
	GG	47 (11.1)	107 (15.3)	0.69 (0.48–0.99)	0.046*
Allele	T	549 (64.6)	852 (60.9)	reference	
	G	301 (35.4)	548 (39.1)	0.85 (0.71–1.02)	0.077
rs7158663					
Co-dominate	GG	224 (52.5)	403 (0.6)	reference	
	GA	170 (39.8)	250 (0.4)	1.22 (0.95–1.58)	0.12
	AA	33 (7.7)	47 (0.1)	1.26 (0.79–2.03)	0.333
Dominate	GG	224 (52.5)	403 (0.6)	reference	
	GA + AA	203 (47.5)	297 (0.4)	1.23 (0.97–1.57)	0.094
Recessive	GG + GA	394 (92.3)	653 (0.9)	reference	
	AA	33 (7.7)	47 (0.1)	1.16 (0.73–1.85)	0.52
Allele	G	618 (72.4)	1056 (75.4)	reference	
	A	236 (27.6)	344 (24.6)	1.17 (0.97–1.42)	0.107

*OR:* odds ratio, *CI:* confidence interval

*The *P* Value < 0.05

### Stratified analysis by age, BMI and menopausal status

Then, we conducted stratified analysis based on age, BMI and menopausal status to further explore their effect on relationship between BC susceptibility and the three SNPs in MEG3. BMI was divided into two levels (BMI < 24 kg/m^2^ and BMI > = 24 kg/m^2^). No association was found between rs11160608, rs7158663 and breast cancer when stratified by age, BMI and menopausal status (Supplemental Table S2). Rs3087918 was related to a reduced susceptibility for women aged <=49 [GG vs. TT: OR (95%CI) = 0.40(0.22–0.73), *P* = 0.02] (Table 3).

**Table 3:** Stratified Analysis of rs3087918 by age, BMI and menopausal status

Group	rs3087918 (Case/Control)			
	TT	TG	GG	TG + GG
**Age**				
< =49	69/93	87/141	19/64	106/205
OR(95%CI)	1.00 (reference)	0.83 (0.55–1.25)	0.40 (0.22–0.73)	0.70 (0.47–1.03)
*P*-value		0.378	0.002*	0.069
> 49	102/166	120/193	28/43	148/236
OR(95%CI)	1.00 (reference)	1.01 (0.72–1.42)	1.06 (0.62–1.81)	1.02 (0.74–1.41)
*P*-value		0.945	0.832	0.901
**BMI (kg/m2)**				
< 24	134/206	147/254	35/74	182/328
OR(95%CI)	1.00 (reference)	0.89 (0.66–1.20)	0.73 (0.46–1.15)	0.85 (0.64–1.13)
*P*-value		0.441	0.171	0.271
> =24	37/53	60/80	12/33	72/113
OR(95%CI)	1.00 (reference)	1.07 (0.63–1.84)	0.52 (0.24–1.14)	0.91 (0.55–1.53)
P-value		0.794	0.100	0.727
**Menstrual-status**				
postmenopausal	114/167	128/201	29/65	157/266
OR(95%CI)	1.00 (reference)	0.93 (0.67–1.29)	0.65 (0.40–1.08)	0.87 (0.64–1.18)
*P*-value		0.675	0.093	0.356
menstruating	57/92	79/133	18/42	97/175
OR(95%CI)	1.00 (reference)	0.96 (0.62–1.48)	0.69 (0.36–1.32)	0.90 (0.59–1.35)
*P*-value		0.848	0.260	0.597

*BMI:* body mass index, *OR:* odds ratio, *CI:* confidence interval

*The *P* Value < 0.05

### Relationship between MEG3 rs3087918 and clinical characteristics of BC

To further explore the effect of rs3087918 loci and clinicopathological information on BC susceptibility, correlation analysis was conducted in the cases group defined by age, BMI, menopausal status, tumor size, metastasis, clinical stage, ER/PR status and Her-2. As showed in Table 4, there is a significant association of the GG genotype with tumor size according to the 95%CI (1.01–3.92), while the *P* value of tumor size is 0.05. In this study, *P* < 0.05 was considered statistically significant. Thus, we considered there was no association found between GG genotype of rs3087918 and tumor size. This is a controversial result that needs further study to clarify. GG and TG + GG genotypes were associated with the over-expression of Her-2 [GG vs. TT: OR (95%CI) = 2.37(1.24–4.63), *P* = 0.010; TG + GG vs. TT: OR (95%CI) = 1.50(1.01–2.24), *P* = 0.045]. We further divided the cases into luminal, Her-2 and triple negative breast cancer (TNBC) groups according to molecular classification. However, we found no association between three SNPs of MEG3 and the different molecular typing states of BC (Supplemental Table S3).

**Table 4:** Relationship between MEG3 rs3087918 and clinical characteristics of cases

rs3087918	TT	TG	GG	TG + GG
**Age**				
> 49/<=49	102/69	120/87	28/19	148/106
OR(95%CI)	1.00 (reference)	0.93 (0.62–1.408)	1.00 (0.52–1.95)	0.94 (0.64–1.40)
*P*-value		0.742	0.993	0.777
**BMI (kg/m**^**2**^**)**				
> =24/< 24	37/134	60/147	12/35	72/182
OR(95%CI)	1.00 (reference)	1.48 (0.92–2.37)	1.24 (0.59–2.63)	1.43 (0.91–2.26)
*P*-value		0.104	0.571	0.120
**Menstrual status**				
yes/no	114/57	128/79	29/18	157/97
OR(95%CI)	1.00 (reference)	0.81 (0.53–1.24)	0.81 (0.42–1.59)	0.81 (0.54–1.21)
*P*-value		0.330	0.526	0.307
**Tumor size (cm)**				
> 2/<=2	85/86	107/100	31/16	138/116
OR(95%CI)	1.00 (reference)	1.08 (0.72–1.62)	1.96 (1.01–3.92)	1.20 (0.82–1.73)
*P*-value		0.701	0.050	0.350
**Metastasis**				
Positive/negtive	93/78	104/103	24/23	128/126
OR(95%CI)	1.00 (reference)	0.85 (0.56–1.27)	0.88 (0.46–1.68)	0.85 (0.58–1.26)
*P*-value		0.422	0.686	0.419
**Clinical Stage**				
III-IV/I-II	51/120	59/148	16/31	75/179
OR(95%CI)	1.00 (reference)	0.94 (0.60–1.47)	1.21 (0.60–2.39)	0.99 (0.65–1.51)
*P*-value		0.778	0.579	0.948
**ER**				
Positive/negtive	115/56	138/69	33/14	171/83
OR(95%CI)	1.00 (reference)	0.97 (0.63–1.50)	1.15 (0.58–2.37)	1.00 (0.66–1.51)
*P*-value		0.904	0.700	0.988
**PR**				
Positive/negtive	94/77	112/95	33/14	145/109
OR(95%CI)	1.00 (reference)	0.97 (0.64–1.45)	1.93 (0.98–3.97)	1.09 (0.74–1.61)
*P*-value		0.867	0.063	0.666
**Her-2**				
Positive/negtive	62/109	90/117	27/20	117/137
OR(95%CI)	1.00 (reference)	1.35 (0.89–2.05)	2.37 (1.24–4.63)	1.50 (1.01–2.24)
*P*-value		0.155	0.01*	0.045*

*BMI:* body mass index, *ER:* estrogen receptor, *PR:* progesterone receptor, *Her-2:* human epidermal growth factor receptor-2, *OR:* odds ratio, *CI:* confidence interval

*The *P* Value < 0.05

### Haplotype analysis of MEG3 SNPs and associations with the risk of BC

To explore the combined effect the three SNPs in MEG3, we performed haplotype analysis by Haploview. The results of the haploid analysis indicated that TCG haplotype may increase the risk of breast cancer compared with the wild haplotype TAG [OR (95%CI) = 2.97(1.66–5.31), *P* < 0.001]. Other haplotypes showed no association with BC (Table 5). The order of the three SNPs was rs3087918, rs11160608 and rs7158663.

**Table 5:** Haplotype analysis of MEG3 rs3087918

Haplotypes	Control (%)	Case (%)	OR (95%)	P
TAG	293 (41.89)	155 (37.44)	reference	–
GCG	206 (29.89)	105 (25.36)	0.96 (0.71–1.31)	0.811
TAA	94 (13.89)	67 (16.18)	1.35 (0.93–1.95)	0.113
GCA	57 (8.89)	33 (7.97)	1.09 (0.68–1.75)	0.707
TCG	21 (3.89)	33 (7.97)	2.97 (1.66–5.31)	< 0.001*

The order of the three SNPs was rs3087918, rs11160608 rs7158663. Haplotypes with frequency less than 0.03 were excluded. *OR:* odds ratio, *CI:* confidence interval

*The *P* Value < 0.05

### The function prediction of the rs3087918 in MEG3

**Fig. 1:**
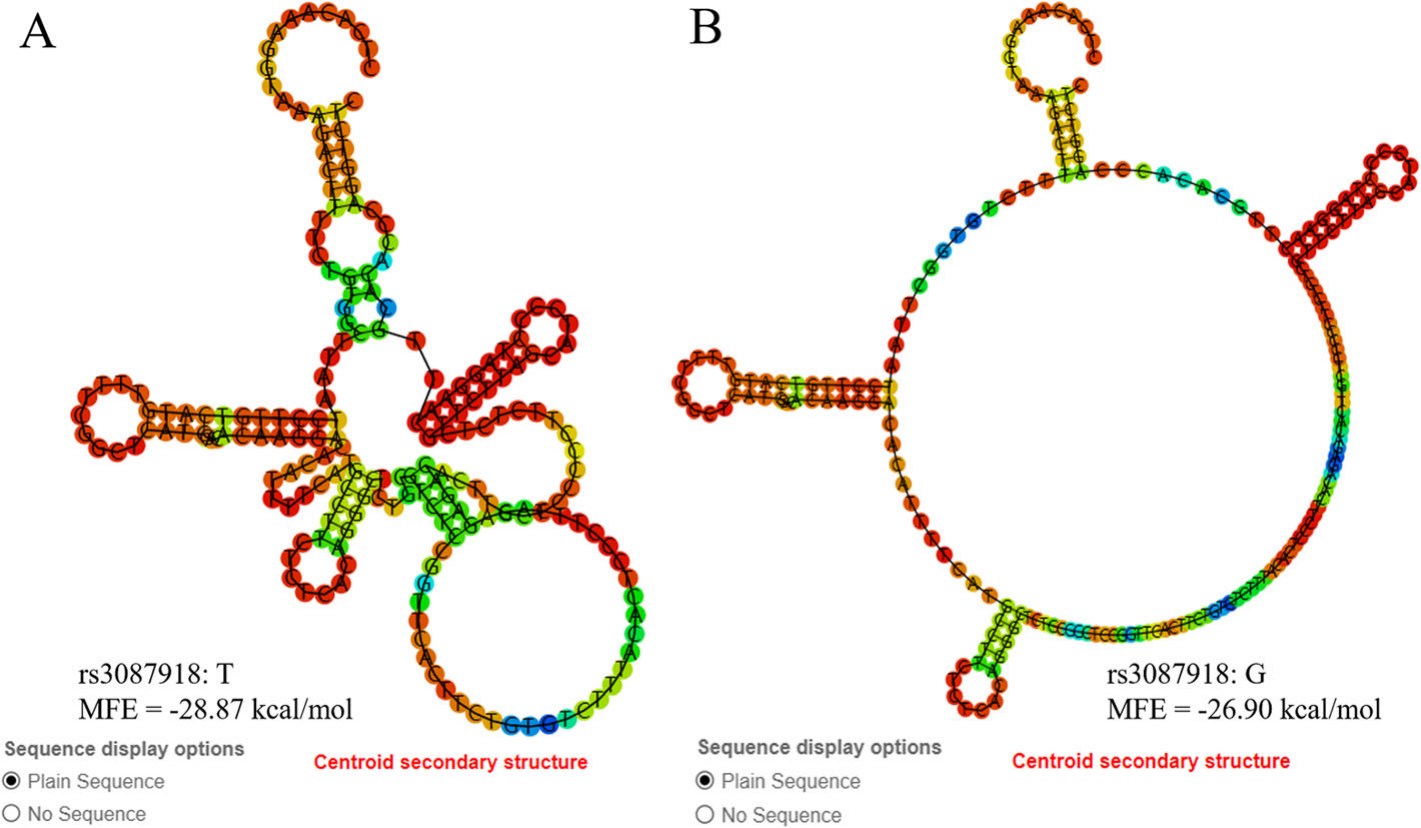
The RNAfold algorithm in silico predicting the impact of rs3087918. MFE: minimum free energy

We used RNAfold (http://rna.tbi.univie.ac.at//cgi-bin/RNAWebSuite/RNAfold.cgi) and LncRNASNP2 (http://bioinfo.life.hust.edu.cn/lncRNASNP/) database to predict the potential function of rs3078918. The centroid secondary structure of rs3087918 was shown in Fig. 1, we learned that mutant allele “G” would significantly change the centroid secondary structure of MEG3. Moreover, its minimum free energy was change from − 28.87 kcal to − 26.90 kcal/mol, which suggests rs3087918 may increase the structural stability of MEG3. The results of LncRNASNP2 indicated that rs3087918 may gain the targets of hsa-miR-1203 to MEG3 (lncRNA ID: NONHSAT039760.2), while loss the target of hsa-miR-139-3p and hsa-miR-5091 to MEG3 (See Supplemental Table S4 and Figure S1).

## Discussion

The occurrence of breast cancer is a result of a long-term complex interaction between individual genetic background and environmental exposure factors. As the most common type of genetic mutation, SNP is of great significance for breast cancer risk, diagnosis, individualized treatment and prognosis prediction. This study is aimed to investigate the association between MEG3 polymorphisms (rs3087918, rs11160608 rs7158663) and breast cancer. Our study recruited 1134 subjects containing 434 breast cancer patients and 700 healthy controls. The results indicated that the mutant homozygous GG of rs3087918 may associated with a decreased risk of BC, especially in females age < = 49. Comparison between case groups showed genotype GG and TG/GG of rs3087918 were correlated with her-2 receptor expression. The results of haplotype analysis for MEG3 showed that compared with wild haploid TAG, TCG haplotype may increase the risk of breast cancer, while other haplotypes were not significantly correlated with breast cancer risk. Furthermore, we found rs3087918 may influence the secondary structure of MEG3 and affect the bind of MEG3 to some miRNAs.

Previous evidences showed that MEG3 was highly expressed in normal tissues such as brain, pituitary, placenta and adrenal gland, and its transcripts can be detected in several human organs including ovary, testes, spleen, pancreas, liver, and mammary gland [7]. However, the expression of MEG3 was lower in various human tumors compared with that in normal human tissues, including breast cancer [24]. MEG3 was recognized as a tumor suppressor deponed on recent researches. In vitro experiments showed that restoring the expression of MEG3 could inhibit cancer cells proliferation and induce their apoptosis [25], and a similar tumor inhibition effect was found in nude mice [16]. MEG3 can also participate in epigenetic regulation of transcripts in the MEG3 region, such as DNA methylation [26, 27], snoRNA/microRNA regulation [28–31]. It is also reported that SNPs in MEG3 gene have an influence on cancer risk. For example, Hou et al. observed a statistically significant increased risk between MEG3 rs11160608 and oral squamous cell carcinoma (OSCC) [24]. And Bayarmaa et al. found MEG3 polymorphisms were related to the chemotherapy response and toxicity of paclitaxel and cisplatin in breast cancer patients [32]. Moreover, Yang et al. found MEG3 rs7158663 have no association with lung cancer, while MEG3 rs4081134 was significantly influence the susceptibility of lung cancer in the Chinese population [33]. In this study, we found MEG3 rs3087918 was associated with a decreased breast cancer risk. We use a database named LncRNASNP2 (http://bioinfo.life.hust.edu.cn/lncRNASNP/) to predict the potential function of rs3087918 on MEG3 gene. The results indicated that rs3087918 may influence MEG3 binding to miRNAs. In detail, rs3087918 may gain the targets of hsa-miR-1203 to MEG3, while loss the target of hsa-miR-139-3p and hsa-miR-5091 to MEG3. A study performed by Tomoyuki Okumura et al. found has-miR-1203 significantly associated with tumor recurrence [34]. Downregulation of has-miR-139-3p could induce cancer cell migration and invasion [35–37], and a pooled analysis proved that high has-miR-139-3p expression was related to a better prognosis for hepatocellular carcinoma [38]. Thus, has-miR-139-3p was attributed as a tumor suppressor [39]. Hsa-miR-5091 was also reported as a biomarker with better prognosis for pancreatic ductal adenocarcinoma [40]. These were coincident with our results that rs3087918 was related to a decreased risk of breast cancer.

To be best of our knowledge, this is the first study to explore the association between MEG3 SNPs (rs3087918, rs11160608 rs7158663) and breast cancer risk. However, there are some potential limitations need to be clarified. First, we failed to consider the potential influence of environmental, lifestyle and other unknow risk factors on our study. Secondly, this is a one center case-control study with a small sample scale, we should not ignore the selective bias. In the future, more complete and larger sample scale study need to accomplish.

## Conclusion

The wild-type homozygous GG of MEG3 rs3087918 was associated with a decreased risk of breast cancer. MEG3 haplotype TCG may increase the risk of breast cancer and it may owe to its effect on the structure and function of MEG3.

## Supplementary information

**Additional file 1: Figure S1.** The prediction results of s3087918 affect the bind of MEG3 to miRNAs. (A) rs3087918 caused has-miR1203 target gain; (B) rs3087918 caused has-miR-139-3p target loss; (C) rs3087918 caused has-miR-5091 target loss. **Table S1.** Primers used for this study. **Table S2.** Stratified Analysis of rs11160608 and rs7158663 by age, BMI and menopausal status. **Table S3.** Association analysis between three SNPs inMEG3 and Molecular typing of breast cancer. **Table S4.** Rs3087918 influence MEG3 binding to miRNAs based on LncRNASNP2 database.

## Data Availability

The datasets generated during and/or analysed during the current study are available from the corresponding author on reasonable request.

## Abbreviations

BC: Breast cancer
MEG3: Maternally expressed gene 3
lncRNA: long non-coding RNA
MAF: Minor allele frequency
HWE: Hardy–Weinberg Equilibrium
BMI: Body mass index
ORs: Odds ratios
CIs: 95% confidence intervals
