# Supplementary material for: LncRNA MEG3 rs3087918 was associated with a decreased breast cancer risk in a Chinese population: a case-control study
Source: BMC Cancer. 2020 Jul 15;20:659. doi: 10.1186/s12885-020-07145-0 (PMC7362410; doi:10.1186/s12885-020-07145-0)
Supplement: Supplementary file 1 — Additional file 1: Figure S1. The prediction results of s3087918 affect the bind of MEG3 to miRNAs. (A) rs3087918 caused has-miR1203 target gain; (B) rs3087918 caused has-miR-139-3p target loss; (C) rs3087918 caused has-miR-5091 target loss. Table S1. Primers used for this study. Table S2. Stratified Analysis of rs11160608 and rs7158663 by age, BMI and menopausal status. Table S3. Association analysis between three SNPs inMEG3 and Molecular typing of breast cancer. Table S4. Rs3087918 influence MEG3 binding to miRNAs based on LncRNASNP2 database. [file 12885_2020_7145_MOESM1_ESM.docx]

**LncRNA MEG3 rs3087918 was associated with a decreased breast cancer risk in a Chinese population: a case-control study**

Yi Zheng^1,2,†^, Meng Wang^3,†^, Shuqian Wang^2,†^, Peng Xu^3^, Yujiao Deng^1,2^, Shuai Lin^3^, Na Li^1,2^, Kang Liu^4^, Yuyao Zhu^1,2^, Zhen Zhai^1,2^, Ying Wu^1,2^, Zhijun Dai^2,3^ and Gaixia Zhu^1^

1. Department of Obstetrics and Gynecology, The Second Affiliated Hospital of Xi’an Jiaotong University, Xi’an 710004, China;

2. Department of Breast Surgery, The First Affiliated Hospital, College of Medicine, Zhejiang University, Hangzhou 310003, China;

3. Department of Oncology, The Second Affiliated Hospital of Xi’an Jiaotong University, Xi’an 710004, China;

4. Department of Hepatobiliary Surgery, The First Affiliated Hospital of Xi'an Jiaotong University, Xi'an 710061, China.

Correspondence to: Gaixia Zhu, Department of Obstetrics and Gynecology, The Second Affiliated Hospital of Xi’an Jiaotong University, Xi’an 710004, China (E-Mail: 306425776@qq.com), or Zhijun Dai, Department of Breast Surgery, The First Affiliated Hospital, College of Medicine, Zhejiang University, Hangzhou 310003, China (E-Mail: dzj0911@126.com).

^†^These authors have contributed equally to this work


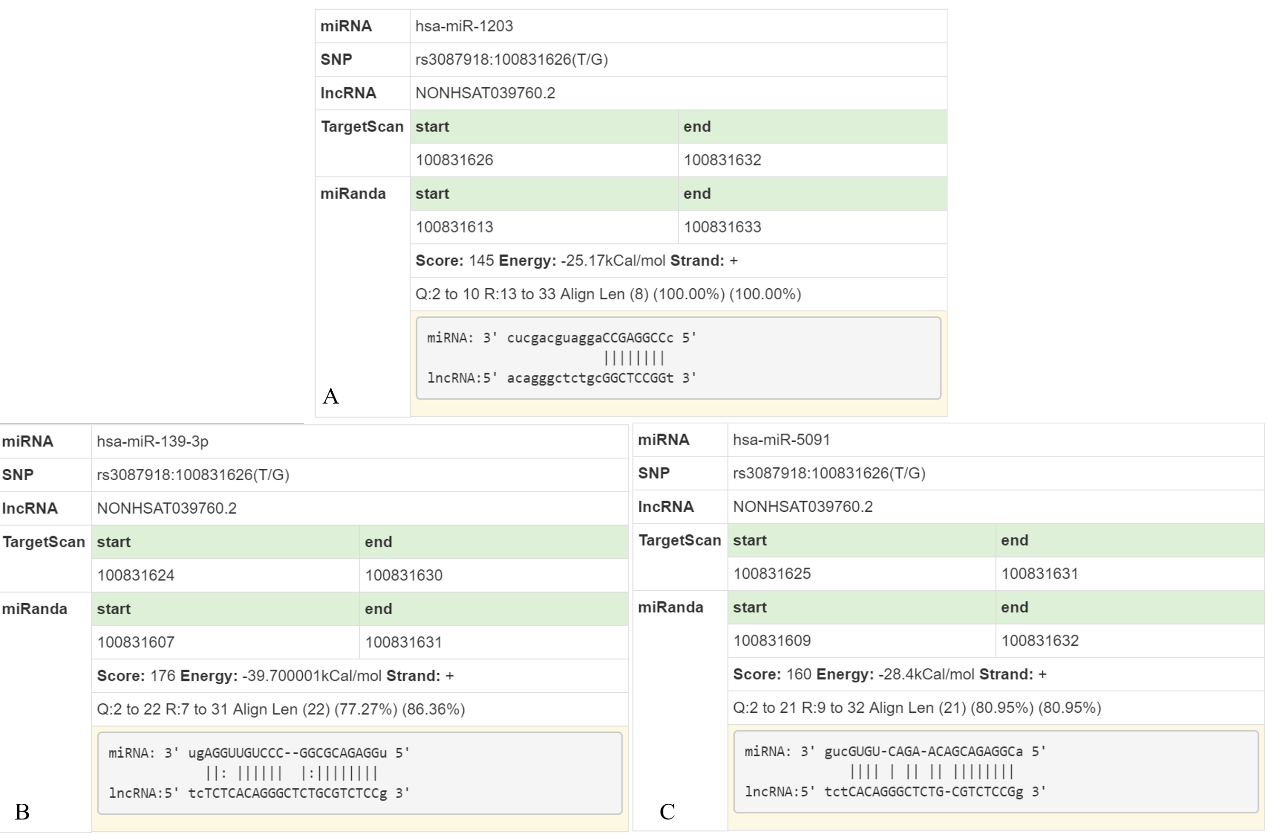


Figure S1. The prediction results of s3087918 affect the bind of MEG3 to miRNAs.

(A) rs3087918 caused has-miR1203 target gain; (B) rs3087918 caused has-miR-139-3p target loss; (C) rs3087918 caused has-miR-5091 target loss.

Table S1. Primers used for this study.

| SNP ID | 1st-PCRP | 2nd-PCRP | UEP_SEQ |
| --- | --- | --- | --- |
| rs11160608 | ACGTTGGATGTATCGCTTTGTGTGTTGCCC | ACGTTGGATGACCATGGTTCATCCGCTTTG | AGGCAATCAATATATAAATAGAAAAAA |
| rs3087918 | ACGTTGGATGAAGGGAGTGTAAAGACACAG | ACGTTGGATGCATGTTTTCGGCTCATGGAC | CTCTCACAGGGCTCTGCG |
| rs7158663 | ACGTTGGATGGTCTGGTACAGAAAGAACCG | ACGTTGGATGTGAGATTCGGGATAGGGTTC | gACAAAAGCCAGAGATAAAAC |

Table S2. Stratified Analysis of rs11160608 and rs7158663 by age, BMI and menopausal status.

| Group | rs11160608 (Case/Control) | | | | rs7158663 (Case/Control) | | | |
| --- | --- | --- | --- | --- | --- | --- | --- | --- |
|  | AA | AC | CC | AC+CC | GG | GA | AA | GA+AA |
| Age |  |  |  |  |  |  |  |  |
| <=49 | 50/102 | 90/143 | 37/53 | 127/196 | 102/177 | 63/102 | 12/9 | 75/121 |
| OR(95%CI) | 1.00 (reference) | 1.28(0.84-1.97) | 1.42(0.83-2.44) | 1.32(0.88-1.98) | 1.00 (reference) | 1.07(0.72-1.60) | 1.10(0.51-2.35) | 1.08(0.74-1.57) |
| P-value |  | 0.253 | 0.198 | 0.177 |  | 0.732 | 0.814 | 0.705 |
| > 49 | 76/125 | 128/198 | 43/79 | 171/277 | 122/226 | 107/148 | 21/28 | 128/176 |
| OR(95%CI) | 1.00 (reference) | 1.06(0.74-1.53) | 0.90(0.56-1.43) | 1.02(0.72-1.43) | 1.00 (reference) | 1.34(0.96-1.87) | 1.39(0.76-2.55) | 1.35(0.98-1.85) |
| P-value |  | 0.739 | 0.643 | 0.931 |  | 0.084 | 0.287 | 0.065 |
| BMI(kg/m2) |  |  |  |  |  |  |  |  |
| <24 | 99/178 | 153/243 | 63/113 | 216/356 | 166/315 | 126/187 | 26/32 | 152/209 |
| OR(95%CI) | 1.00 (reference) | 1.13(0.82-1.56) | 1.00(0.68-1.49) | 1.09(0.81-1.47) | 1.00 (reference) | 1.28(0.95-1.72) | 1.54(0.89-2.67) | 1.32(1.00-1.74) |
| P-value |  | 0.445 | 0.99 | 0.567 |  | 0.101 | 0.121 | 0.053 |
| >=24 | 27/49 | 65/98 | 17/19 | 82/117 | 58/88 | 44/63 | 7/15 | 51/78 |
| OR(95%CI) | 1.00 (reference) | 1.20(0.68-2.12) | 1.62(0.73-3.63) | 1.27(0.74-2.20) | 1.00 (reference) | 1.06(0.64-1.76) | 0.71(0.27-1.84) | 0.99(0.61-1.61) |
| P-value |  | 0.52 | 0.237 | 0.389 |  | 0.823 | 0.478 | 0.974 |
| Menstrual-status |  |  |  |  |  |  |  |  |
| postmenopausal | 82/156 | 138/203 | 49/74 | 187/277 | 134/232 | 114/168 | 24/33 | 138/201 |
| OR(95%CI) | 1.00 (reference) | 1.29(0.92-1.82) | 1.26(0.80-1.97) | 1.28(0.93-1.78) | 1.00 (reference) | 1.18(0.85-1.62) | 1.26(0.71-2.22) | 1.19(0.88-1.61) |
| P-value |  | 0.142 | 0.313 | 0.131 |  | 0.322 | 0.425 | 0.264 |
| menstruating | 44/71 | 80/138 | 31/58 | 111/196 | 90/171 | 56/82 | 9/14 | 65/96 |
| OR(95%CI) | 1.00 (reference) | 0.94(0.59-1.49) | 0.86(0.49-1.53) | 0.91(0.59-1.42) | 1.00 (reference) | 1.30(0.85-1.99) | 1.22(0.51-2.93) | 1.29(0.86-1.93) |
| P-value |  | 0.779 | 0.614 | 0.69 |  | 0.229 | 0.654 | 0.223 |

BMI: body mass index; OR: odds ratio; CI: confidence interval.

Table S3. Association analysis between three SNPs inMEG3 and Molecular typing of breast cancer.

| SNPs  genetic model | Luminal | | |  | Her-2 | | |  | TNBC | | |
| --- | --- | --- | --- | --- | --- | --- | --- | --- | --- | --- | --- |
|  | Yes/No | OR(95%CI) | *P* |  | Yes/No | OR(95%CI) | *P* |  | Yes/No | OR(95%CI) | *P* |
| rs3087918 |  |  |  |  |  |  |  |  |  |  |  |
| TT | 121/50 | reference |  |  | 26/145 | reference |  |  | 24/147 | reference |  |
| TG | 141/66 | 0.88(0.57-1.37) | 0.579 |  | 29/178 | 0.91(0.51-1.61) | 0.743 |  | 37/170 | 1.33(0.76-2.33) | 0.313 |
| GG | 36/11 | 1.35(0.64-2.87) | 0.430 |  | 6/41 | 0.82(0.32-2.12) | 0.676 |  | 5/52 | 0.59(0.21-1.62) | 0.302 |
| TG+GG | 177/77 | 0.95(0.62-1.45) | 0.812 |  | 35/219 | 0.89(0.52-1.54) | 0.681 |  | 42/212 | 1.21(0.70-2.09) | 0.485 |
| rs11160608 |  |  |  |  |  |  |  |  |  |  |  |
| AA | 86/40 | reference |  |  | 24/102 | reference |  |  | 16/110 | reference |  |
| AC | 147/71 | 0.96(0.60-1.54) | 0.875 |  | 36/182 | 0.84(0.48-1.49) | 0.551 |  | 35/183 | 1.32(0.70-2.49) | 0.399 |
| CC | 60/20 | 1.40(0.74-2.62) | 0.299 |  | 8/72 | 0.47(0.20-1.11) | 0.081 |  | 12/68 | 1.21(0.54-2.72) | 0.639 |
| AC+CC | 207/91 | 1.06(0.68-1.66) | 0.805 |  | 44/254 | 0.74(0.43-1.27) | 0.272 |  | 47/251 | 1.29(0.70-2.37) | 0.416 |
| rs7158663 |  |  |  |  |  |  |  |  |  |  |  |
| GG | 157/67 | reference |  |  | 37/187 | reference |  |  | 30/194 | reference |  |
| GA | 110/60 | 0.78(0.51-1.20) | 0.257 |  | 28/142 | 1.00(0.58-1.71) | 0.990 |  | 32/138 | 1.50(0.87-2.58) | 0.143 |
| AA | 26/7 | 1.59(0.66-3.83) | 0.303 |  | 4/29 | 0.70(0.23-2.10) | 0.620 |  | 3/30 | 0.65(0.19-2.25) | 0.780 |
| GA+AA | 136/67 | 0.87(0.58-1.30) | 0.491 |  | 32/171 | 0.95(0.56-1.58) | 0.830 |  | 35/168 | 1.35(0.79-2.29) | 0.269 |

TNBC: triple negative breast cancer; OR: odds ratio; CI: confidence interval.

Table S4. Rs3087918 influence MEG3 binding to miRNAs based on LncRNASNP2 database.

| miRNA ID | lncRNA | SNP ID | Energy (kCal/Mol) | Binding Start (TargetScan) | Binding End (TargetScan) | Binding Start (miRanda) | Binding End (miRanda) | Function of miRNA |
| --- | --- | --- | --- | --- | --- | --- | --- | --- |
| has-miR-1203 | MEG3 | rs3087918 | -25.17 | 100831626 | 100831632 | 100831613 | 100831633 | Target gain |
| hsa-miR-139-3p | NONHSAT039760.2 | rs3087918 | -39.700001 | 100831624 | 100831630 | 100831607 | 100831631 | Target loss |
| hsa-miR-5091 | NONHSAT039760.2 | rs3087918 | -28.4 | 100831625 | 100831631 | 100831609 | 100831632 | Target loss |
